# Inequalities and Chronic Non-Communicable Diseases in Brazil

**DOI:** 10.1590/SS2237-9622202200016.especial

**Published:** 2022-07-08

**Authors:** Fernando C. Wehrmeister, Andrea T. Wendt, Luciana M.V. Sardinha

**Affiliations:** 1 Universidade Federal de Pelotas, Programa de Pós-Graduação em Epidemiologia, Pelotas, RS, Brazil; 2 Vital Strategies Brasil, São Paulo, SP, Brazil

Brazil currently has an epidemiological scenario with a predominance of chronic noncommunicable diseases (NCDs) such as hypertension, diabetes *mellitus* and cancers, which have well-known risk factors, including smoking, unhealthy diet, abusive consumption of alcoholic beverages, in addition to low physical activity. In 2017, about three out of four deaths were attributed to NCDs in Brazil.^1^ Even though the proportional mortality is high, from 1990 to 2017 there was a 35% reduction in deaths resulting from NCDs in the country.^1^ However, national estimates of health indicators can hide important inequalities.

The Sustainable Development Goals (SDGs), agreed upon in 2015, set as a target, in addition to the global reduction of mortality from NCDs, the reduction of inequalities associated with such deaths.^2^ Despite the positive result in the reduction of mortality from NCDs in the country in the nearly three decades mentioned, with prospects of reaching the SDG target,^3^ such a reduction was of 48.9% (95%CI -50.8;-46.8) in the Federal District, while stability was observed in Rio Grande do Norte (-2.8%; 95%CI -8.3;3.2)^1^.

Faced with this scenario, it is essential to highlight the importance of monitoring, either of health outcomes or their inequalities. In this context, Brazil has a tradition of collecting information on health-related outcomes through surveys and by means of its health information systems. An example is the National Health Survey (PNS), a population-based survey conducted for the first time in 2013. In the chronic diseases module, a series of information on chronic diseases, health problems and use of health services are collected. This kind of information provides a very comprehensive and reliable overview of the Brazilian population health.

Using data from the two editions of the PNS, a study on multimorbidity in people aged between 18 and 59 years showed that the presence of two or more morbidities increased in the country and was inversely related to schooling: the prevalence of multimorbidity in less educated individuals was around 10 percentages points higher than in more educated individuals.^4^ It is also worth noting that inequalities are not only related to individual determinants (e.g. age, race/skin color, education and wealth), but in a country such as Brazil, with continental dimensions, the region and area of residence of individuals, as well as the context in which they live, can reveal important differences in health estimates. Additionally, depending on the indicator studied, the intersectionality approach (overlapping of social strata) can highlight groups who are more vulnerable.

**Figure 1:**
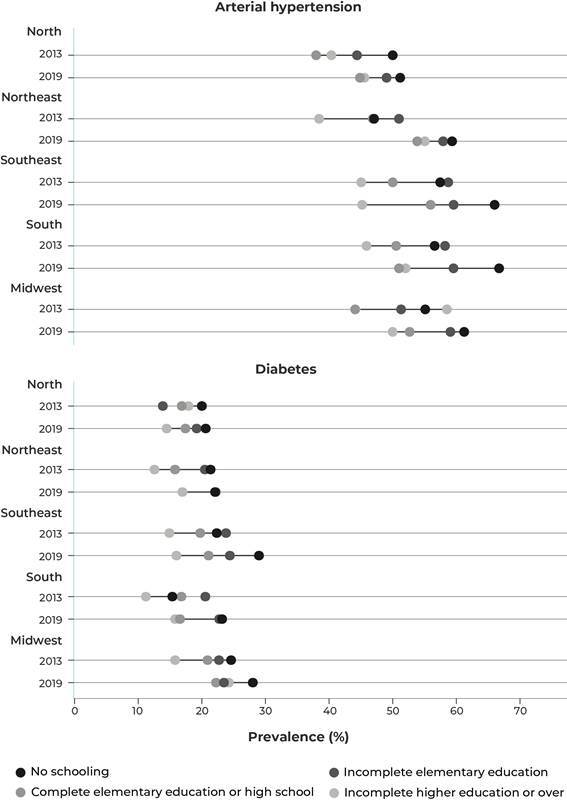
Prevalence of arterial hypertension and diabetes, in elderly Brazilians aged 60 and over, by region and education, National Health Survey, 2013 and 2019

Source: National Health Survey, 2013 and 2019.

Figure 1 shows inequalities in the prevalence of arterial hypertension and diabetes *mellitus* by schooling, in the elderly (≥ 60 years) residing in each of the five Brazilian macro-regions. In this illustration, we can see the application of the intersectionality approach -, in this specific case, the combination of region and schooling strata. In general, for all regions and for both diseases, a pattern is observed where the less educated individuals are more affected by the diseases than the more educated individuals. This type of visualization makes it possible to monitor the changes that have occurred in the prevalence of hypertension and diabetes with a greater focus on patterns of inequality. 

Specifically with regard to intersectionality, the most evident example of the increase in inequalities in these diseases can be seen in the Southeast region. The prevalence of arterial hypertension, for example, remained stable, between 2013 and 2019, in the most educated individuals, around 44%, while among the less educated, it increased from 56% to 65%. This increase in the most vulnerable group led to a substantial increase in the observed inequalities. It should be noted that the same pattern is observed for diabetes *mellitus*, both at a given time and over time for the Southeast region. On the other hand, in the North and Northeast regions, there are indications that a decrease in the differences between more and less educated individuals has occurred, despite the increase in the prevalence of the outcome. Therefore, this type of approach reveals not only in which groups there is an increase/decrease in the health indicator of interest, but also if that change is uniform across the population, or if any stratum presents an important social disadvantage. Observing such patterns of inequality enables managers to better plan and monitor public actions and policies aimed precisely at tackling NCDs.

Another important aspect in the study of inequalities is its relationship with inequities. While inequality is the measurable part of disparities between population groups, that is, what we can measure based on information from health surveys or using health information systems, inequity is a theoretical concept, difficult to measure and subject to value judgment.^2^ ^,^ ^5^ ^,^ ^6^

In order to characterize an inequity in health, attention must be paid to some aspects. The first one is to identify a systematization in the patterns of inequalities. In the example of Figure 1, with a few exceptions, in all regions, both diabetes *mellitus* and arterial hypertension were more prevalent in the least educated. The second aspect is related to how such a pattern was produced. There are patterns that are biologically produced, while others have strong social determination. In the example in question, the less educated tend to experience worse living and working conditions, besides being more exposed to risk factors for chronic diseases, such as physical inactivity, inadequate diet, abusive consumption of alcohol, tobacco use, among others. Lastly, in addition to these two aspects, an inequality is considered an inequity when it is understood as unfair and also avoidable. To those interested in the topic of inequality a value judgment to understand whether what is being observed can be characterized as an inequity is in order.

Monitoring health indicators, either of diseases or health coverage, is essential for keeping the health of the population under observation. It is clear that aggregate estimates have their value, but they end up hiding important inequalities in population subgroups. In addition to the obvious constant investment in research and improvement of health systems, health surveys and services should be encouraged to collect information on different dimensions of inequality: skin color, ethnicity, gender, migration status, region of residence, among so many other possibilities. The SDGs themselves already suggest that reliable, current data should be available that allow a broader view at health inequalities. 

In the current scenario of Brazilian health, with the fiscal austerity policies implemented in 2016 and the problems in facing health crises, such as the COVID-19 pandemic, health research focused on identifying those individuals who are "being left behind" becomes even more relevant. A careful look is essential so that we can collectively face the great burden that NCDs impose on society and on health services. This effort could be more effective if all the actors involved (politicians, managers, researchers and the civil society) adopted joint and coordinated actions aimed not only to reduce NCDs, but also to reduce inequalities associated with these health outcomes.
